# Amino Acid Derivatives of Ligustrazine-Oleanolic Acid as New Cytotoxic Agents

**DOI:** 10.3390/molecules191118215

**Published:** 2014-11-07

**Authors:** Fuhao Chu, Xin Xu, Guoliang Li, Shun Gu, Kuo Xu, Yan Gong, Bing Xu, Mina Wang, Huazheng Zhang, Yuzhong Zhang, Penglong Wang, Haimin Lei

**Affiliations:** 1 School of Chinese Pharmacy, Beijing University of Chinese Medicine, Beijing 100102, China; E-Mails: chufhao@163.com (F.C.); xu.xin@vip.126.com (X.X.); liguoliangsx@163.com (G.L.); xukuoworld@126.com (K.X.); gongyan90@163.com (Y.G.); weichenxubing@126.com (B.X.); 2 Key Laboratory for Neurodegenerative Diseases of Ministry of Education, Xuanwu Hospital of Capital Medical University, Beijing 100053, China; E-Mail: gszy2003@163.com; 3 School of Basic Medicine, Beijing University of Chinese Medicine, Beijing 100029, China; E-Mails: wangmina2013@126.com (M.W.); zhanghuazheng1109@163.com (H.Z.); zyz100102@126.com (Y.Z.)

**Keywords:** amino acid, ligustrazine-oleanolic acid, anticancer, ClogP, low toxicity, Giemsa and DAPI staining

## Abstract

A series of novel ligustrazine-oleanolic acid (TOA) derivatives were designed, and synthesized by conjugating amino acids to the 3-hydroxy group of TOA by ester bonds. Their cytotoxicity was evaluated on four cancer cell lines (HepG2, HT-29, Hela and BGC-823) by standard MTT assays. The ClogP values were calculated by means of computer simulation, and logP values of both 3*β-*glycine ester olean-12-en-28-oic acid-3,5,6-trimethylpyrazin-2-methyl ester (**6a**) and TOA were determined using a shake flask-ultraviolet spectrophotometry method. It was found that **6a** and the 3*β*-l-lysine ester-**6g** not only displayed good cytotoxicity (IC_50_ < 3.5 μM) but also possessed better hydrophilicity than TOA. Moreover, **6a** (IC_50_ = 4.884 μM) had lower nephrotoxicity than both **6g** (IC_50_ = 2.310 μM) and cisplatin (CDDP, IC_50_ = 3.691 μM) on MDCK cells. Combining Giemsa and DAPI staining, it was further verified that **6a** could induce HepG2 apoptosis via nuclei fragmentation and had lower nephrotoxicity. In addition, the structure-activity relationships of these derivatives are briefly discussed.

## 1. Introduction

**Figure 1:**
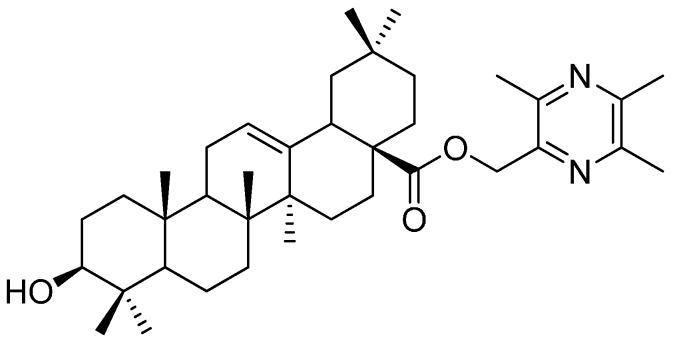
The structure of the anticancer lead compound TOA.

Based on the principle of chemical combination, the attempt to discover lead compounds from Traditional Chinese Medicines (TCMs) has drawn considerable attentions [1,2,3,4,5,6]. There are potential advantages in having such agents with complementary pharmacological activities in the form of a single chemical entity [2]. In the earlier studies in this field, several ligustrazine derivatives had been designed, synthesized and biologically evaluated [3,4,5,6]. Thereafter, a novel anticancer lead compound 3*β-*hydroxyolea-12-en-28-oic acid-3,5,6-trimethylpyrazin-2-methyl ester (TOA, C_38_H_58_O_3_N_2_, Figure 1) was screened [6,7,8,9,10,11]. TOA was synthesized by conjugating the effective antitumor ingredients ligustrazine (TMP) and oleanolic acid (OA). It exhibited promising anticancer effects *in vitro* [6] and prevented the expression of nuclear transcription factor NF-κB/p65 and COX-2 in S180 mice [7]. In addition, the acute toxicity tests verified that the LD_50_ value of TOA exceeded 6.0 g/kg via gavage in mice. However, the shortcoming of TOA’s poor hydrophilicity limited its oral bioavailability. In our previous study, solid dispersions and microemulsion systems of TOA were developed to enhance the bioactivity of TOA, and they displayed favorable pharmacokinetic profiles *in vivo*. The TOA microemulsion formulation showed 28-fold and 4-fold higher maximum concentration (C_max_) than the pure drug and solid dispersion, respectively [10,11]. 

As essential nutrients of the human body, amino acids have favorable hydrophilicity. Numerous studies have showed that by introducing amino acid groups into the structures of insoluble drugs, the hydrophilicity and the bioactivity could be enhanced [12,13,14,15,16,17,18,19,20,21,22]. In addition, some studies have indicated that low aqueous-solubility drugs conjugated with amino acids or dipeptides could be selectively identified and taken up by certain transporters, thus significantly improving their membrane permeability and cytotoxicity [22,23]. 

In this paper, twelve different amino acids were selected for conjugation to the 3-hydroxy moiety of TOA by ester bond formation. Their cytotoxicity was then evaluated on four cancer cell lines, including the human hepatoma cell line HepG2, human colorectal cancer cell line HT-29, human cervical cancer cell line Hela and human gastric cancer cell lines BGC-823, by standard thiazolyl blue (MTT) assays. In the study, the antineoplastic drug cisplatin (CDDP) was selected as the positive control. Due to its nephrotoxicity in clinical use, the Madin-Darby canine kidney cell line (MDCK) was used to test the toxicity of the TOA-amino acid derivatives and CDDP. The ClogP values were calculated by means of computer simulation. logP values of both **6a** and TOA were determined using a shake flask-ultraviolet spectrophotometry method. Model cellular morphological and nuclear damage studies were carried out by Giemsa and DAPI staining, respectively. In addition, the structure-activity relationships of the new TOA-amino acid derivatives are briefly discussed.

## 2. Results and Discussion

### 2.1. Chemistry

**Scheme 1:**
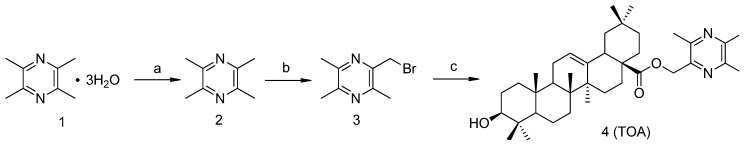
Synthesis route to TOA.

Compound 3 (TMP-Br) was synthesized by the treatment of anhydrous tetramethylpyrazine (TMP) with N-bromosuccinimide (NBS) [24]. The product was used in the next step without further purification. Compound **4** (TOA) was obtained through the formation of an ester bond between compound **3** and OA after stirring for 1.5 h at room temperature under alkaline conditions in dry tetrahydrofuran (THF) (Scheme 1).

*Reagents and conditions*: (a) Benzene, reflux, 10 h, 100.0%; (b) CCl_4_, NBS , hν, reflux, 12 h, 70.0%; (c) OA, THF, K_2_CO_3_, reflux, 1.5 h, 57.0%.

**Scheme 2:**
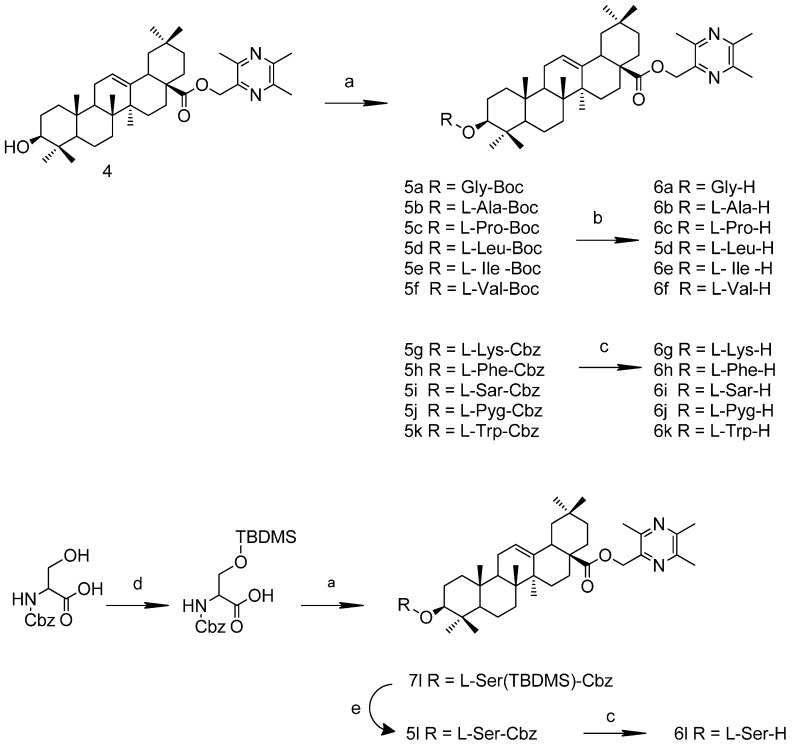
Synthesis routes to TOA-amino acid derivatives.

To avoid the formation of by-products, the amino acid groups were protected with t-butyloxy- carbonyl (Boc*-*) or benzoyloxycarbonyl groups (Cbz*-*). The general synthesis of compounds **6a**–**6l** was conducted according to Scheme 2. Ester condensation reactions were catalyzed by 1-(3-dimethylaminopropyl)-3-ethylcarbodiimide hydrochloride (EDCI) and 4-dimethylaminopyridine (DMAP). The removal of the Boc*-* groups was conducted in 3 M HCl/ethyl acetate solution, and Cbz- groups were de-protected with H_2_, Pd/C (or Pd(OH)_2_/C). Before esterification with TOA, the hydroxyl group of Cbz*-*L*-*serine was protected with TBDMS-Cl; the TBDMS group of the intermediate was de-protected with TBAF to produce **6l**. The structures of the new compounds that had not been reported previously were elucidated by HRMS and NMR spectroscopy.

### 2.2. Biological Activities

The cytotoxicity of TOA-amino acid derivatives was evaluated on HepG2, HT-29, Hela and BGC-823 cancer cells using the MTT assay. In addition their toxicity evaluation was carried out on MDCK cells. As shown in Table 1, most of the synthesized compounds exhibited preferable cytotoxicity. Compounds **6a**, **6b**, **6c**, **6g** and **6l** demonstrated much better cytotoxicity (IC_50_ < 8.0 μM) than TOA on all cancer cell lines. In particular, **6a** and **6g** showed much better cytotoxicity (IC_50_ < 3.5 μM) against the four cell lines than the others. Moreover, **6a** (IC_50_ = 4.884 μM) had lower nephrotoxicity than both **6g** (IC_50_ = 2.310 μM) and CDDP (IC_50_ = 3.691 μM) on MDCK cells.

*Reagents and conditions*: (a) EDCI, DAMP, r.t.12 h, 45%–90%; (b) 3 M HCl/ethyl acetate solution, r.t.1 h, 90%; (c) 10% Pd/C or Pd(OH)_2_/C, H_2_, r.t.12 h, 96%; (d) TBDMS-Cl, imidazole, DMF, r.t.12 h; (e) TBAF, dry THF, ice-salt bath, 0.5 h.

In addition, it was observed that introducing different types of amino acids on the 3-hydroxy group of TOA significantly different cytotoxicity resulted. It was worth noting that cytotoxicity showed a negative correlation with the length of amino acids’ branched carbon chain, in the order **6a** > **6b** > **6f** > **6e** > **6d**. The cytotoxicity of cyclic secondary amines was better than that of cyclic acylamino derivatives, such as **6c** > **6j**. Increasing the number of amino groups might enhance the activity. For instance, **6g** containing two amino groups had strong cytotoxicity (IC_50_ < 2.5 μM) on both cancer and MDCK cells. 

**Table 1:** Anti-proliferative effects and ClogP values of TOA-amino acid derivatives.

Compound	IC_50_ Values (μM)					ClogP
	HepG2	HT-29	Hela	BGC-823	MDCK	
**CDDP**	2.856	6.112	9.838	6.819	3.691	-^b^
**TOA**	21.45	-^a^	8.683	-	-	8.125
**6a**	1.999	2.498	3.256	3.062	4.884	7.433
**6b**	2.322	4.770	7.690	4.474	5.125	7.835
**6c**	2.391	3.213	6.754	4.976	5.034	8.590
**6d**	-	-	-	-	-	8.763
**6e**	5.969	9.830	-	-	-	8.750
**6f**	4.257	5.956	-	9.522	9.785	8.479
**6g**	2.270	2.347	2.383	2.481	2.310	7.343
**6h**	8.216	9.503	-	-	8.119	8.831
**6i**	10.22	16.85	12.61	16.04	15.78	8.346
**6j**	5.785	5.480	11.47	10.73	12.62	8.117
**6k**	11.44	-	-	10.01	10.82	8.895
**6l**	4.783	4.829	5.217	4.898	3.122	6.860

The derivatives were evaluated on human hepatoma cell lines (HepG2), human colorectal cancer cell lines (HT-29), women cervical cancer cell lines (Hela) and human gastric cancer cell lines (BGC-823) using MTT assay, as well as their toxicity evaluation was carried out on Madin-Darby canine kidney cell lines (MDCK). -^a^ meant IC_50_ > 25 μM, the compounds’ effects were considered to be weaker than TOA and not evaluated. Data are derived from three independent experiments. -^b^ means the ClogP value was not calculated, due to structural differences between CDDP and others.

### 2.3. Computer Simulation of ClogP and Determination of logP

Lipophilicity (ClogP) is used in rational drug design as a key factor related to the cell transmembrane transport or other biological processes [25,26,27]. ClogP values can either be calculated experimentally [28,29,30] or predicted by means of commercially available programs [31,32,33,34,35]. In this work, ClogP values of the TOA-amino acid derivatives were predicted using Sybyl-X 2.0 (Tripos, Certara Inc., St. Louis, MO, USA) [5]. As shown in Table 1, **6a**, **6b**, **6g**, **6j** and **6l** possessed lower values compared to the ClogP value of TOA. It found that smaller amino acids, and polar or basic amino acids could decrease the derivatives’ ClogP values. To determine the octanol-water partition coefficient (logP), a shake flask-ultraviolet spectrophotometry method [36] was used to determine the concentration of **6a** and TOA in the water phase and organic phase at pH 6.8 with *n*-caprylic alcohol-water as the simulation system. It determined that the logP values of **6a** and TOA were 4.30 and 6.11, respectively. This indicated that by conjugating amino acids the logP value could be decreased and the hydrophilicity improved, which was in accordance with previous studies [12,13,21,22]. However, the better activity connected with the increases of these amino acid conjugates’ solubility is worthy of further study.

### 2.4. Changes in Cellular Morphologies [37]

Apoptosis has been considered a major mechanism of chemotherapy-induced cell death, and the drug cytotoxicity often elicits cellular morphology changes [38], including loss of intercellular contacts, membrane damage and nuclear fragmentation. These characteristic changes are usually considered as the hallmarks of apoptosis [39]. 

#### 2.4.1. Geimsa Staining

**Figure 2:**
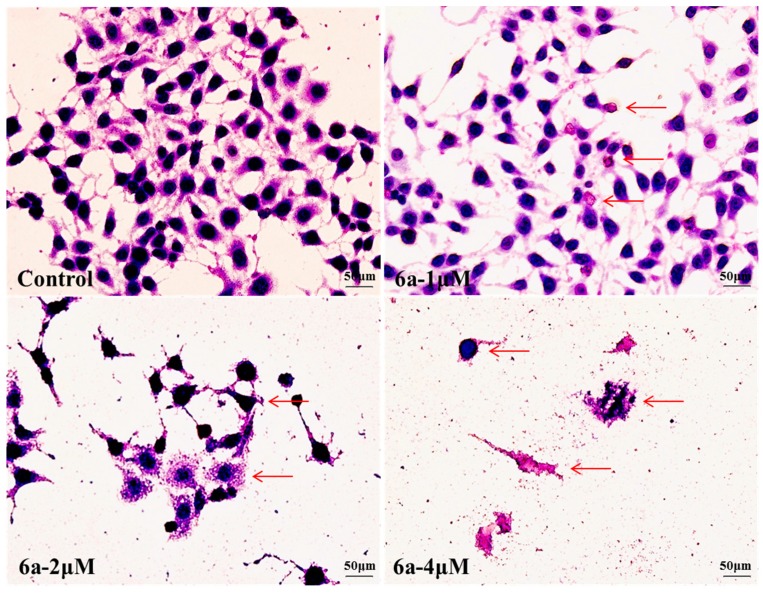
Morphological changes of HepG2 cells after treatment with 0, 1, 2 and 4 μM of **6a** was observed under inverted phase-contrast microscope at a magnification of 400×. Cells showed changes in cell morphology, included loss of intercellular contacts, lysis of nuclei, increased cell debris and membrane damage (arrows) with increasing doses of **6a** when compared to the control cells. Data were derived from three independent experiments.

To confirm the apoptopic morphological changes induced by **6a**, HepG2 cells were treated with 0, 1, 2 and 4 μM of **6a** for 72 h and then Giemsa staining was performed. The morphology changes observed in treated cells compared to control cells under inverted phase-contrast microscope at a magnification of 400×, included loss of intercellular contacts, lysis of nuclei, increased cell debris and membrane damage (marked by arrows in Figure 2). Meanwhile, we could clearly observe that the morphological changes of HepG2 cells significantly increased with increasing concentration of **6a**. There was no complete cell morphology when cells were treated with 2 and 4 μM of **6a**.

**Figure 3:**
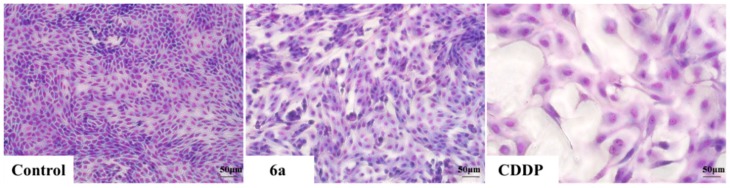
Morphology of MDCK cells after treatment with **6a** and CDDP was observed under a microscope in the same multiples (200×). Data were derived from three independent experiments.

To test the agents’ nephrotoxicity, MDCK cells were exposed to 2 μM of compound **6a** and CDDP for 72 h. Then Giemsa staining was performed on model cells. Changes were observed in the CDDP- treated groups under inverted phase-contrast microscope at a magnification of 200×, including less density, larger volume, lighter nuclear staining and cellular atypia. However, no significant morphological changes were observed in cells treated with **6a** and control cells (Figure 3). By combining Giemsa staining, we found that **6a** could induce HepG2 apoptosis and had lower nephrotoxicity than CDDP. 

#### 2.4.2. DAPI Staining

Apoptosis can be differentiated from necrosis by their characteristic nuclear changes. DAPI is a nuclear stain which is observed as blue fluorescence when excited under fluorescence microscope [39]. 

**Figure 4:**
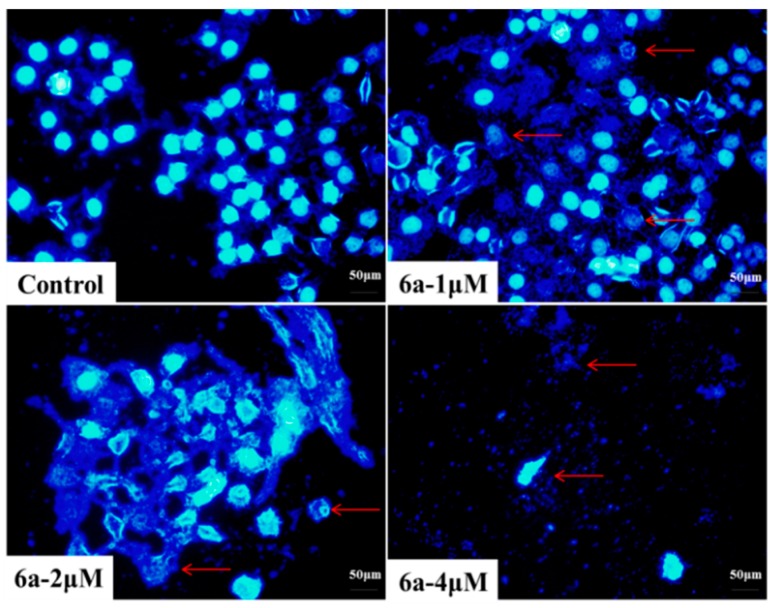
Nuclear fragmentation of HepG2 cells induced by compound **6a** with different doses (0, 1, 2 and 4 μM), which was observed using inverted phase-contrast microscope at a magnification of 400× with an excitation wavelength of 470 nm. Data were derived from three independent experiments.

In our present study, HepG2 cells were treated with 0, 1, 2 and 4 μM of **6a** for 72 h and then DAPI staining was performed. Control cells (0 μM) showed intact cell bodies with clear round nuclei, while treated cells clearly showed condensed chromatin, nuclear fragmentation and weak fluorescence compared to the control cells (Figure 4). Meanwhile, we could clearly observe that nuclear fragmentation of HepG2 cells increased significantly with increasing concentration of **6a**. Thus, DAPI staining indicated that **6a** could induce HepG2 apoptosis via nuclear fragmentation.

## 3. Experimental Section 

### 3.1. General Information

Reactions were monitored by TLC using silica gel coated aluminum sheets (Qingdao Haiyang Chemical Co., Qingdao, China) and visualized in UV light (254 nm). ^1^H-NMR and ^13^C-NMR assays were recorded on a Bruker AVANCE 500 NMR spectrometer (Fällanden, Switzerland) and chemical shifts are reported in form of δ (ppm). Deuterated chloroform or deuterated dimethylsulfoxide was served as the solvent (Beijing InnoChem Science and Technology Co., Ltd., Beijing, China). HRMS spectra were performed on High-resolution ESI mass spectrum (Solarix 9.4T, Bruker, Germany). Samples were freeze-dried using the vacuum freeze dryer (Beijing Boyikang Co., Ltd., Beijing, China). Melting points were measured at a rate of 5 °C/min using an X-5 micro melting point apparatus (Beijing Tech Instrument Co., Ltd., Beijing, China). The specific rotation of the synthesized compounds was measured using P-1020 polarimeter (Jasco, Tokyo, Japan). DAHZ-300 versatile constant temperature bath oscillator (Taicang experimental plant, Jiangsu, China) and TU-1810 UV-Vis spectrophotometer (Beijing Purkinje General Instrument Co., Ltd., Beijing, China) were used in determination of the oil-water partition coefficients. Cellular morphologies were observed using an inverted fluorescence microscope (Olympus IX71, Tokyo, Japan). Silica-gel column chromatography was performed using 200–300 mesh silica gel. The yields were calculated based on the last step reaction. All solvents and chemicals used were analytical or high-performance liquid chromatography grade.

### 3.2. Chemistry

*2-(Bromomethyl)-3,5,6-Trimethylpyrazine* (**3**)

Compound **3** was prepared according to our previously reported method [24]. The crude product, with 70% purity, was not purified further because of its strong mucous membrane irritation.

*3β-Hydroxyolea-12-en-28-oic acid-3,5,6-trimethylpyrazine-2-methyl Ester* (TOA, **4**)

The crude product was obtained according to a reported method [6]. After further purification by silica-gel column chromatography, the purity was improved to more than 95%, and the product could be used directly for the later reactions. 

#### 3.2.1. General Synthesis of the TOA-Amino Acid Derivatives **6a**–**6f**

The corresponding t-butyloxycarbonyl-protected amino acids (0.38 mmol), EDCI (0.50 mmol) and DMAP (0.025 mmol) were dissolved in dry DCM, then compound **4** (0.25 mmol) was added and the reaction solution was stirred at room temperature overnight. The crude product was extracted with CH_2_Cl_2_. After drying the organic layer over anhydrous Na_2_SO_4_ and evaporating the solvent under vacuum, the crude product was purified by flash chromatography with dichloromethane-methanol (20:1) as eluent. The product was stirred in ethyl acetate with 3 M hydrochloric acid for 1 h. The reaction solution was then evaporated with vacuum at 30 °C. The crude products were neutralized with saturated sodium bicarbonate solution and extracted with dichloromethane. After separating by flash chromatography with dichloromethane-methanol (20:1) as eluent, the products were lyophilized. 

*3β-Glycine ester olean-12-en-28-oic acid-3,5,6-trimethylpyrazin-2-methyl ester* (**6a**). White solid, m.p. 96.0–97.6 °C, ${\left[\alpha \right]}_{D}^{25}$ = +63.8 (*c* 1.0, MeOH), yield: 64%. ^1^H-NMR (CDCl_3_) δ (ppm): ^1^H-NMR (CDCl_3_) δ (ppm): 0.55, 0.85, 0.86, 0.89, 1.11 (s, 15H, 5×-CH_3_, methyl of OA), 0.90 (brs, 6H, 2×-CH_3_, methyl of OA), 2.24 (brs, 2H, -NH_2_), 1.00–2.50 (23H, methine- and methylene- of OA), 2.49, 2.51, 2.55 (s, each, 3H, 3×-CH_3_, methyl of TMP), 2.86 (m, 1H, -CH=CCH-), 3.48 (s, 2H,-NH_2_CH_2_-), 4.55 (m, 1H, -OCOCH-), 5.12, 5.19 (d, each, *J* = 10 Hz, 1H, -OCH_2_-), 5.24 (brs, 1H, -CH=C-). ^13^C-NMR (CDCl_3_) δ (ppm): 15.7, 16.8, 16.9, 18.2, 23.0, 23.4, 23.6, 25.8, 27.6, 28.1, 29.7, 30.7, 32.4, 32.6, 33.1, 33.8, 36.9, 37.8, 38.1, 39.2, 41.3, 41.6, 43.7 (-CH_2_NH_2_), 45.9, 47.5, 55.3, 64.9, 81.9 (-OCOCH-), 122.3 (-CH=C-), 143.6 (-CH=C-), 173.4 (-CO-), 177.2 (-CO-), pyrazine ring: 20.6 (-CH_3_), 21.4 (-CH_3_), 21.7 (-CH_3_), 64.9 (-COCH_2_-), 145.4, 148.8, 149.2, 151.0. HRMS (ESI) *m/z*: 648.47410 [M+H]^+^, calcd for C_40_H_6__2_N_3_O_4_: 648.4662.

*3β-l-Alanine ester olean-12-en-28-oic acid-3,5,6-trimethylpyrazin-2-methyl ester* (**6b**). White solid, m.p. 89.7–92.1 °C, ${\left[\alpha \right]}_{D}^{25}$ = +30.5 (*c* 1.0, MeOH), yield: 56%. ^1^H-NMR (CDCl_3_) δ (ppm): 0.54, 0.89, 1.11 (s, each, 3H, 3×-CH_3_, methyl of OA), 0.87 (brs, 6H, 2×-CH_3_, methyl of OA), 0.91 (brs, 6H, 2×-CH_3_, methyl of OA), 1.00–2.50 (25H, methyl of *L-alanine*, methine- and methylene- of OA), 2.49, 2.51, 2.55 (s, each, 3H, 3×-CH_3_, methyl of TMP), 2.74 (brs, 2H, -NH_2_), 2.86 (m, 1H, -CH=CCH-), 3.58 (s, 1H, -NH_2_CH-), 4.53 (m, 1H, -OCOCH-), 5.12, 5.19 (d, each, *J* = 10 Hz, 1H, -OCH_2_-), 5.24 (brs, 1H, -CH=C-). ^13^C-NMR (CDCl_3_) δ (ppm): 15.7, 16.7, 16.8, 18.2, 23.0, 23.5, 23.7, 25.8, 27.6, 28.1, 28.2, 30.7, 32.4, 32.6, 33.1, 33.8, 36.9, 37.8, 38.0, 38.1, 39.2, 41.3, 41.6, 45.9, 46.9, 47.5, 55.3, 55.4 (-CHNH_2_), 81.9 (-OCOCH-), 122.3 (-CH=C-), 143.6 (-CH=C-), 173.4 (-CO-), 177.2 (-CO-), pyrazine ring: 20.6 (-CH_3_), 21.4 (-CH_3_), 21.7 (-CH_3_), 64.9 (-COCH_2_-), 145.4, 148.8, 149.2, 151.0. HRMS (ESI) *m/z*: 662.48967 [M+H]^+^, calcd for C_41_H_6__4_N_3_O_4_: 662.48186.

*3β-l-Proline ester olean-12-en-28-oic acid-3,5,6-trimethylpyrazin-2-methyl ester* (**6c**). White solid, m.p. 89.6–91.3 °C, ${\left[\alpha \right]}_{D}^{25}$ = +29.0 (*c* 1.0, MeOH), yield: 51%. ^1^H-NMR (CDCl_3_) δ (ppm): 0.54, 0.86, 0.87, 0.89, 1.11 (s, each, 3H, 5×-CH_3_, methyl of OA), 0.91 (brs, 6H, 2×-CH_3_, methyl of OA), 1.00–2.50 (26H, methylene- of l-proline, methine- and methylene- of OA), 2.49, 2.51, 2.55 (s, each, 3H, 3×-CH_3_, methyl of TMP), 2.86 (m, 1H, -CH=CCH-), 2.95, 3.10 (m, 2H, -NHCH_2_-), 3.74 (brs, 1H, -NH-), 3.81 (m, 1H, -NHCH-), 4.55 (m, 1H, -OCOCH-), 5.12, 5.19 (d, each, *J* = 10.0 Hz, 1H, -OCH_2_-), 5.24 (brs, 1H, -CH=C-). ^13^C-NMR (CDCl_3_) δ (ppm): 15.7, 16.9, 18.1, 23.0, 23.4, 23.6, 23.7, 25.4, 25.8, 27.6, 28.1, 29.7, 30.5, 30.7, 32.4, 32.6, 33.1, 33.8, 36.9, 37.8, 38.1, 39.2, 41.3, 41.6, 45.9, 46.9, 47.0 (-NHCH_2_-), 47.5, 55.3, 60.0 (-NHCH-), 81.9 (-OCOCH-), 122.3 (-CH=C-), 143.6 (-CH=C-), 174.7 (-CO-), 177.2 (-CO-), pyrazine ring: 20.6 (-CH_3_), 21.4 (-CH_3_), 21.7 (-CH_3_), 64.9 (-COCH_2_-), 145.4, 148.8, 149.2, 151.0. HRMS (ESI) *m/z*: 688.50525[M+H]^+^, calcd for C_43_H_6__6_N_3_O_4_: 688.49751. 

*3β-l-Leucine ester olean-12-en-28-oic acid-3,5,6-trimethylpyrazin-2-methyl ester* (**6d**). White solid, m.p. 89.6–91.6 °C, ${\left[\alpha \right]}_{D}^{25}$ = +42.0 (*c* 1.0, MeOH), yield: 49%. ^1^H-NMR (CDCl_3_) δ (ppm): 0.54, 0.89, 1.10 (s, each, 3H, 3×-CH_3_, methyl of OA), 0.87 (brs, 6H, 2×-CH_3_, methyl of OA), 0.91 (brs, 6H, 2×-CH_3_, methyl of OA), 0.96 (m, 6H, 2×-CH_3_, methyl of *L-leucine*), 1.00–2.50 (27H, methine- and methylene- of l-leucine and OA), 2.49, 2.51, 2.54 (s, each, 3H, 3×-CH_3_, methyl of TMP), 2.86 (m, 1H, -CH=CCH-), 3.51 (m, 1H, -CHNH_2_), 4.53 (m, 1H, -OCOCH-), 5.12, 5.19 (d, each, *J* = 10.0 Hz, 1H, -OCH_2_-), 5.24 (brs, 1H, -CH=C-). ^13^C-NMR (CDCl_3_) δ (ppm): 15.7, 16.8, 18.2, 21.8, 23.0, 23.1, 23.4, 23.5, 23.6, 24.8, 25.8, 27.6, 28.1, 29.7, 30.7, 32.4, 32.6, 33.1, 33.8, 36.9, 37.8, 38.1, 39.2, 41.3, 41.6, 43.7, 45.9, 46.9, 47.5, 53.3 (-CHNH_2_), 55.3, 81.9 (-OCOCH-), 122.3 (-CH=C-), 143.6 (-CH=C-), 173.4(-CO-), 177.2 (-CO-), pyrazine ring: 20.6 (-CH_3_), 21.4 (-CH_3_), 21.7 (-CH_3_), 64.9 (-COCH_2_-), 145.4, 148.8, 149.2, 151.0. HRMS (ESI) *m/z*: 704.53694 [M+H]^+^, calcd for C_44_H_70_N_3_ O_4_: 704.52881.

*3β-l-Isoleucine ester olean-12-en-28-oic acid-3,5,6-trimethylpyrazin-2-methyl ester* (**6e**). White solid, m.p. 89.8–90.7 °C, ${\left[\alpha \right]}_{D}^{25}$ = +44.8 (*c* 1.0, MeOH), yield: 49%. ^1^H-NMR (CDCl_3_) δ (ppm): 0.55, 0.89, 1.11 (s, each, 3H, 3×-CH_3_, methyl of OA), 0.88 (brs, 6H, 2×-CH_3_, methyl of OA), 0.91 (brs, 6H, 2×-CH_3_, methyl of OA), 1.01 (m, 3H, -CH_3_, methyl of L*-*isoleucine), 1.00–2.50 (30H, methyl-, methine- and methylene- of l-isoleucine, methine- and methylene- of OA), 2.49, 2.51, 2.54 (s, each, 3H, 3×-CH_3_, methyl of TMP), 2.87 (m, 1H, -CH=CCH-), 3.42 (m, 1H, -CHNH_2_), 4.53 (m, 1H, -OCOCH-), 5.12, 5.19 (d, each, *J* = 10.0 Hz, 1H, -OCH_2_-), 5.24 (brs, 1H, -CH=C-). ^13^C-NMR (CDCl_3_) δ (ppm): 11.7, 15.3, 15.9, 16.8, 16.9, 18.2, 23.1, 23.4, 23.6, 23.8, 24.4, 25.8, 27.6, 28.1, 30.7, 32.4, 32.6, 33.1, 33.8, 36.9, 37.7, 38.1, 38.7, 39.2, 41.3, 41.6, 45.9, 46.9, 47.5, 55.3, 59.7 (-CHNH_2_), 81.9 (-OCOCH-), 122.3 (-CH=C-), 143.6 (-CH=C-), 173.5 (-CO-), 177.2 (-CO-), pyrazine ring: 20.6 (-CH_3_), 21.4 (-CH_3_), 21.7 (-CH_3_), 64.9 (-COCH_2_-), 145.4, 148.8, 149.4, 151.1. HRMS (ESI) *m/z*: 704.53668 [M+H]^+^, calcd for C_44_H_70_N_3_O_4_: 704.52881.

*3β-l-Valine ester olean-12-en-28-oic acid-3,5,6-trimethylpyrazin-2-methyl ester* (**6f**). White solid, m.p. 89.5–91.0 °C, ${\left[\alpha \right]}_{D}^{25}$ = +29.3 (*c* 1.0, MeOH), yield: 57%. ^1^H-NMR (CDCl_3_) δ (ppm): 0.55, 0.89, 1.11 (s, each, 3H, 3×-CH_3_, methyl of OA), 0.88 (brs, 6H, 2×-CH_3_, methyl of OA), 0.91 (brs, 6H, 2×-CH_3_, methyl of OA), 1.00–2.50 (31H, methyl- and methine- of l-valine, methine- and methylene- of OA), 2.49, 2.51, 2.54 (s, each, 3H, 3×-CH_3_, methyl of TMP), 2.87 (m, 1H, -CH=CCH-), 3.31 (m, 1H, -CHNH_2_), 4.53 (m, 1H, -OCOCH-), 5.12, 5.19 (d, each, *J* = 10.0 Hz, 1H, -OCH_2_-), 5.24 (brs, 1H, -CH=C-). ^13^C-NMR (CDCl_3_) δ (ppm): 15.3, 16.7, 16.8, 16.9, 18.2, 19.7, 23.1, 23.4, 23.6, 23.7, 25.8, 27.6, 28.1, 30.7, 31.7, 32.4, 32.6, 33.1, 33.8, 36.9, 37.7, 38.1, 39.2, 41.3, 41.6, 45.9, 46.9, 47.5, 55.3, 60.4(-CHNH_2_), 81.9 (-OCOCH-), 122.3 (-CH=C-), 143.6 (-CH=C-), 173.5(-CO-), 177.2 (-CO-), pyrazine ring: 20.6 (-CH_3_), 21.4 (-CH_3_), 21.7 (-CH_3_), 64.9 (-COCH_2_-), 145.4, 148.8, 149.4, 151.1. HRMS (ESI) *m/z*: 690.52101 [M+H]^+^, calcd for C_43_H_6__8_N_3_O_4_: 690.51316. 

#### 3.2.2. General Synthesis of the TOA-Amino Acid Derivatives **6g**–**6k**

The corresponding benzoyloxycarbonyl-protected amino acids (0.38 mmol), EDCI (0.50 mmol) and DMAP (0.025 mmol) were dissolved in dry DCM, then compound **4** (0.25 mmol) was added and the reaction solution was stirred at room temperature overnight. The crude product was extracted with CH_2_Cl_2_. After drying the organic layer over anhydrous Na_2_SO_4_ and evaporating the solvent under vacuum, the crude product was purified by flash chromatography with dichloromethane-methanol (20:1) as eluent. The crude product and a little 10% Pd/C or Pd(OH)_2_/C were stirred in methanol under a hydrogen atmosphere overnight. The reaction solution was filtered and evaporated with vacuum. After separation by flash chromatography with dichloromethane-methanol (20:1) as eluent, the product was lyophilized. 

*3β-l-Lysine ester olean-12-en-28-oic acid-3,5,6-trimethylpyrazin-2-methyl ester* (**6g**). White solid, m.p. 87.5–88.2 °C, ${\left[\alpha \right]}_{D}^{25}$ = +156.0 (*c* 1.0, MeOH), yield: 69%. ^1^H-NMR (CDCl_3_) δ (ppm): 0.55, 0.89, 1.11 (s, each, 3H, 3×-CH_3_, methyl of OA), 0.88 (brs, 6H, 2×-CH_3_, methyl of OA), 0.91 (brs, 6H, 2×-CH_3_, methyl of OA), 1.00–2.50 (32H, methylene- of l-lysine, methine- and methylene- of OA), 2.49, 2.51, 2.54 (s, each, 3H, 3×-CH_3_, methyl of TMP), 2.75 (m, 2H, -CH_2_NH_2_), 2.87 (m, 1H, -CH=CCH-), 3.44 (m, 1H, -CHNH_2_), 4.52 (m, 1H, -OCOCH-), 5.12, 5.19 (d, each, *J* = 10.0 Hz, 1H, -OCH_2_-), 5.24 (brs, 1H, -CH=C-). ^13^C-NMR (CDCl_3_) δ (ppm): 15.3, 16.8, 16.9, 18.2, 22.9, 23.1, 23.4, 23.5, 23.6, 25.8, 27.6, 28.1, 30.7, 31.0, 32.4, 32.6, 33.1, 33.8, 34.4, 36.9, 37.7, 38.1, 39.2, 41.4, 41.6, 41.8, 45.9, 46.9, 47.5, 54.6 (-CHNH_2_), 55.3, 81.9 (-OCOCH-), 122.3 (-CH=C-), 143.6 (-CH=C-), 175.3 (-CO-), 177.2 (-CO-), pyrazine ring: 20.6 (-CH_3_), 21.4 (-CH_3_), 21.7 (-CH_3_), 64.9 (-OCOCH_2_-), 145.4, 148.8, 149.2, 151.0. HRMS (ESI) m/z: 719.54488 [M+H]^+^, calcd for C_44_H_7__1_N_4_O_4_: 719.53971.

*3β-l-Phenylalanine ester olean-12-en-28-oic acid-3,5,6-trimethylpyrazin-2-methyl ester* (**6h**). White solid, m.p. 82.4–83.8 °C, ${\left[\alpha \right]}_{D}^{25}$ = +63.3 (*c* 1.0, MeOH), yield: 60%. ^1^H-NMR (CDCl_3_) δ (ppm): 0.54, 0.79, 0.84, 0.89, 0.90, 0.91, 1.11 (s, each, 3H, 7×-CH_3_, methyl of OA), 1.00–2.50 (24H, methine- and methylene- of OA), 2.49, 2.51, 2.54 (s, each, 3H, 3×-CH_3_, methyl of TMP), 2.87 (m, 1H, -CH=CCH-), 3.16 (m, 2H, -CH_2_CHNH_2_), 3.76 (m, 1H, -CHNH_2_), 4.53 (m, 1H, -OCOCH-), 5.12, 5.19 (d, each, *J* = 10.0 Hz, 1H, -OCH_2_-), 5.24 (brs, 1H, -CH=C-), 7.22, 7.23, 7.29, 7.30, 7.31 (m, 5H, -C_6_H_5_). ^13^C-NMR (CDCl_3_) δ (ppm): 15.3, 16.8, 16.9, 18.2, 23.1, 23.4, 23.5, 23.6, 25.9, 27.6, 28.1, 30.7, 32.4, 32.6, 33.1, 33.8, 36.9, 37.7, 38.1, 39.2, 41.0, 41.3, 41.7, 45.9, 46.9, 47.5, 55.3, 56.1 (-CHNH_2_), 81.6 (-OCOCH-), 122.3 (-CH=C-), 143.6 (-CH=C-), 175.3 (-CO-), 177.2 (-CO-), benzene ring: 126.8, 128.5, 128.6, 129.3, 129.4, 137.2, pyrazine ring: 20.6 (-CH_3_), 21.4 (-CH_3_), 21.7 (-CH_3_), 64.9 (-OCOCH_2_-), 145.4, 148.8, 149.2, 151.0. HRMS (ESI) *m/z*: 738.52094 [M+H]^+^, calcd for C_47_H_6__8_N_3_O_4_: 738.51316.

*3β-l-Sarcosine ester olean-12-en-28-oic acid-3,5,6-trimethylpyrazin-2-methyl ester* (**6i**). White solid, m.p. 81.9–82.5 °C, ${\left[\alpha \right]}_{D}^{25}$ = +48.8 (*c* 1.0, MeOH), yield: 55%. ^1^H-NMR (CDCl_3_) δ (ppm): 0.55, 0.86, 0.87, 0.89, 1.11 (s, each, 3H, 5×-CH_3_, methyl of OA), 0.91 (brs, 6H, 2×-CH_3_, methyl of OA), 2.45 (s, 3H, -NHCH_3_), 1.00–2.50 (23H, methine- and methylene- of OA), 2.49, 2.51, 2.54 (s, each, 3H, 3×-CH_3_, methyl of TMP), 2.87 (m, 1H, -CH=CCH-), 3.37 (s, 2H, -CH_2_NH-), 4.57 (m, 1H, -OCOCH-), 5.12, 5.19 (d, each, *J* = 10.0 Hz, 1H, -OCH_2_-), 5.24 (brs, 1H, -CH=C-). ^13^C-NMR (CDCl_3_) δ (ppm): 15.4, 16.7, 16.8, 18.3, 23.1, 23.4, 23.6, 23.6, 25.8, 27.6, 28.1, 30.7, 32.4, 32.6, 33.1, 33.9, 36.0 (-NHCH_3_), 36.9, 37.7, 38.1, 39.2, 41.3, 41.6, 45.9, 46.9, 47.5, 52.9 (-CH_2_NH-), 55.3, 81.5 (-OCOCH-), 122.3 (-CH=C-), 143.6 (-CH=C-), 172.0(-CO-), 177.2 (-CO-), pyrazine ring: 20.6 (-CH_3_), 21.4 (-CH_3_), 21.7 (-CH_3_), 64.9 (-OCOCH_2_-), 145.4, 148.8, 149.2, 151.0. HRMS (ESI) *m/z*: 662.48967 [M+H]^+^, calcd for C_41_H_6__4_N_3_O_4_: 662.48186.

*3β-l-Pyroglutamate ester olean-12-en-28-oic acid-3,5,6-trimethylpyrazin-2-methyl ester* (**6j**). White solid, m.p. 124.6–125.8 °C, ${\left[\alpha \right]}_{D}^{25}$ = +35.6 (*c* 1.0, MeOH), yield: 45%. ^1^H-NMR (CDCl_3_) δ (ppm): 0.55, 0.89, 1.11 (s, each, 3H, 3×-CH_3_, methyl of OA), 0.88 (brs, 6H, 2×-CH_3_, methyl of OA), 0.91 (brs, 6H, 2×-CH_3_, methyl of OA), 1.00–2.50 (27H, methylene- of l-pyroglutamate, methine- and methylene- of OA), 2.50, 2.52, 2.56 (s, each, 3H, 3×-CH_3_, methyl of TMP), 2.87 (m, 1H, -CH=CCH-), 4.25 (m, 1H, -CHNH-), 4.57 (m, 1H, -OCOCH-), 5.12, 5.19 (d, each, *J* = 10.0 Hz, 1H, -OCH_2_-), 5.24 (brs, 1H, -CH=C-). ^13^C-NMR (CDCl_3_) δ (ppm): 15.3, 16.8, 16.8, 18.2, 23.1, 23.4, 23.5, 23.8, 25.1, 25.9, 27.7, 28.1, 29.3, 30.7, 32.4, 32.6, 33.1, 33.9, 36.9, 37.9, 38.1, 39.2, 41.3, 41.9, 45.9, 46.9, 47.5, 55.3, 55.7 (-CHNH-), 82.5 (-OCOCH-), 122.3 (-CH=C-), 143.6 (-CH=C-), 171.7 (-CO-), 177.2 (-CO-), 177.7 (-CONH-), pyrazine ring: 20.6 (-CH_3_), 21.4 (-CH_3_), 21.7 (-CH_3_), 64.9 (-OCOCH_2_-), 145.4, 148.8, 149.2, 150.9. HRMS (ESI) *m/z*: 702.48461 [M+H]^+^, calcd for C_43_H_6__4_N_3_O_5_: 702.47677.

*3β-l-Tryptophan ester olean-12-en-28-oic acid-3,5,6-trimethylpyrazin-2-methyl ester* (**6k**). White solid, m.p. 206.9–207.7 °C, ${\left[\alpha \right]}_{D}^{25}$ = +39.3 (*c* 1.0, MeOH), yield: 43%. ^1^H-NMR (DMSO-d_6_) δ (ppm): 0.35, 0.60, 0.69, 0.81, 1.06 (s, each, 3H, 5×-CH_3_, methyl of OA), 0.87 (brs, 6H, 2×-CH_3_, methyl of OA), 2.40, 2.42, 2.46 (s, each, 3H, 3×-CH_3_, methyl of TMP), 1.00–2.50 (24H, methine- and methylene- of OA). 2.76 (m, 1H, -CH=CCH-), 2.96, 3.12 (m, 2H, -CH_2_CHNH_2_), 3.75 (m, 1H, -CHNH_2_), 4.35 (m, 1H, -OCOCH-), 5.09, 5.10, (d, each, *J* = 10.0 Hz, 1H, -OCH_2_-), 5.11 (brs, 1H, -CH=C-), indole ring: 6.97, 7.06 (m, each, 1H), 7.14 (brs, -CHNH-), 7.34, 7.53 (d, each, J = 10.0 Hz, 1H), 10.9 (s, 1H, -NH). ^13^C-NMR (DMSO-d_6_) δ (ppm): 14.9, 16.3, 16.4, 17.6, 22.6, 22.8, 23.0, 23.3, 25.4, 26.9, 27.4, 30.1, 30.3, 32.0, 32.1, 32.7, 33.1, 36.3, 37.1, 37.4, 38.7, 40.9, 41.2, 45.4, 46.2, 46.7, 54.4, 54.9 (-CHNH_2_), 80.6 (-OCOCH-), 121.8 (-CH=C-), 143.2 (-CH=C-), 173.3 (-CO-), 176.1 (-CO-), indole ring: 109.2, 111.4, 118.2, 118.3, 120.9, 123.8, 127.1, 136.2, pyrazine ring: 20.1 (-CH_3_), 20.9 (-CH_3_), 21.2 (-CH_3_), 64.2 (-OCOCH_2_-), 145.0, 148.2, 148.7, 150.6. HRMS (ESI) *m/z*: 777.53162 [M+H]^+^, calcd for C_49_H_6__9_N_4_O_4_: 777.52406. 

#### 3.2.3. Synthesis of the TOA-Amino Acid Derivative **6l**

Cbz-l-serine (1.0 mmol), TBDMS-Cl (1.2 mmol) and imidazole (2.0 mmol) were stirred in DMF at room temperature overnight. The crude product was extracted with CH_2_Cl_2_. After drying the organic layer over anhydrous Na_2_SO_4_ and evaporating the solvent under vacuum, the crude product was purified by flash chromatography with dichloromethane-methanol (20:1) as eluent. The crude product (0.50 mmol), EDCI (0.50 mmol) and DMAP (0.025 mmol) were dissolved in dry DCM, then compound **4** (0.25 mmol) was added and the reaction solution was stirred at room temperature overnight. The crude product was extracted with CH_2_Cl_2_. After drying the organic layer over anhydrous Na_2_SO_4_ and evaporating the solvent under vacuum, the crude product was purified by flash chromatography with dichloromethane-methanol (20:1) as eluent. The crude product and 1.5 equivalents of TBAF were stirred in THF for about 0.5 h in an ice-bath. The crude product was extracted with CH_2_Cl_2_. After drying the organic layer over anhydrous Na_2_SO_4_ and evaporating the solvent under vacuum, the crude product was purified by flash chromatography with dichloromethane-methanol (20:1) as eluent. The crude product and a little 10% Pd/C or Pd(OH)_2_/C were stirred in methanol under a hydrogen atmosphere overnight. The crude product was extracted with CH_2_Cl_2_. After drying the organic layer over anhydrous Na_2_SO_4_ and evaporating the solvent under vacuum, the crude product was purified by flash chromatography with dichloromethane-methanol (20:1) as eluent. The product was lyophilized.

*3β-l-Serine ester olean-12-en-28-oic acid-3,5,6-trimethylpyrazin-2-methyl ester* (**6l**). White solid, m.p. 107.1–108.5 °C, ${\left[\alpha \right]}_{D}^{25}$ = +55.5 (*c* 1.0, MeOH), yield: 36%. ^1^H-NMR (CDCl_3_) δ (ppm): 0.55, 0.89, 1.11 (s, each, 3H, 3×-CH_3_), 0.87 (brs, 6H, 2×-CH_3_, methyl of OA), 0.91 (brs, 6H, 2×-CH_3_, methyl of OA), 2.17 (s, 1H, -OH), 1.00–2.50 (25H, methine- and methylene- of OA), 2.49, 2.51, 2.54 (s, each, 3H, 3×-CH_3_, methyl of TMP), 2.71 (m, 2H, -CH_2_OH), 2.87 (m, 1H, -CH=CCH-), 4.54 (m, 1H, -OCOCH-), 5.12, 5.19 (d, each, *J* = 10.0 Hz, 1H, -OCH_2_-), 5.24 (brs, 1H, -CH=C-). ^13^C-NMR (CDCl_3_) δ (ppm): 15.7, 16.8, 16.9, 18.2, 23.0, 23.5, 23.7, 23.8, 25.8, 27.6, 28.1, 30.7, 32.4, 32.6, 33.1, 33.8, 36.9, 37.8, 38.0, 39.2, 41.3, 41.6, 45.9, 46.9, 47.5, 55.3, 56.1 (-CHNH_2_), 63.7 (-CH_2_OH), 81.9 (-OCOCH-), 122.3 (-CH=C-), 143.6 (-CH=C-), 173.4 (-CO-), 177.2 (-CO-), pyrazine ring: 20.6 (-CH_3_), 21.4 (-CH_3_), 21.7 (-CH_3_), 64.9 (-OCOCH_2_-), 145.4, 148.8, 149.2, 151.0. HRMS (ESI) *m/z*: 678.48175 [M+H]^+^, calcd for C_41_H_6__4_N_3_O_5_: 678.47677. 

### 3.3. Bio-Evaluation Methods

#### 3.3.1. Cytotoxicity Evaluation

The cytotoxicity of these compounds was tested on five cells lines by the standard MTT assay. The human cancer cells lines (HepG2, HT-29, Hela, BGC-823) and MDCK cells were provided by the Chinese Academy of Medical Sciences Peking Union Medical College. The tumor cells were cultured in RPMI 1640 medium supplemented with 10% (v/v) fetal bovine serum and 1% (v/v) penicillin-streptomycin (Thermo Technologies, New York, NY, USA) at 37 °C in a humidified atmosphere with 5% CO_2_. The growing tumor cells at a density 3 × 10^3^ cells/mL were exposed to various concentrations of the tested drugs and incubated in a 96-well microtiter plate for 72 h (37 °C, 5% CO_2_). After MTT solution (20 μL, 5 mg/mL) was added to each well, the plate was incubated for a further 4 h. Then the media was removed. Formazan crystals were dissolved with DMSO (150 μL). After mixing well, the absorbance was quantified at 490 nm with a BIORAD 550 spectrophotometer (Bio-rad Life Science Development Ltd., Beijing, China). Wells containing no drugs were used to be blanks. The IC_50_ values were defined as the concentration of compounds that produced a 50% reduction of surviving cells and calculated using the Logit-method. Tumor cell growth inhibitory rate was calculated in the following Equation (1):

% inhibition = (1 − Sample group OD/Control group OD) × 100%(1)

#### 3.3.2. Giemsa Staining [37]

HepG2 cells in logarithmic growth phase were cultured in 6-well plates for 24 h at 37 °C in a humidified atmosphere with 5% CO_2_. And then each group was treated with different concentrations of compound **6a** for 72 h. The cell culture medium was discarded. And the cells were washed twice with PBS, kept in PBS/ethanol (1:1) for 2 min, fixated in ethanol for 10 min. After removing the ethanol, the cells were stained with Giemsa stain for 2 min and observed under inverted phase-contrast microscope at a magnification of 400×. MDCK cells in logarithmic growth phase were cultured in 6-well plates for 24 h at 37 °C in a humidified atmosphere with 5% CO_2_. The cells were treated with 2 μM of compound **6a** and CDDP for 72 h. The same operation was experimented as described above, and the cells were observed under inverted phase-contrast microscope at a magnification of 200×.

#### 3.3.3. DAPI Staining [40,41]

HepG2 cells in logarithmic growth phase were allowed to grow in 6-well plates for 24 h at 37 °C in a humidified atmosphere with 5% CO_2_. And each group was treated with different concentrations of compound **6a** for 72 h. Cell culture medium was discarded and the cells were washed twice with PBS. The cells were fixed with 4% paraformaldehyde (pH 7.4) for 15 min and then washed twice with PBS. With an excitation wavelength of 470 nm, 4',6-diamidino-2-phenylindole (DAPI, 600 nM, Molecular Probes/Invitrogen Life Technologies, Carlsbad, CA, USA) staining was then performed for 2 min and nuclear fragments were observed using inverted phase-contrast microscope at a magnification of 400×.

## 4. Conclusions

In summary, a series of novel TOA-amino acid derivatives were synthesized by conjugating amino acids to the 3-hydroxy group of TOA by ester bonds. The target products were characterized by spectroscopic data. Their cytotoxicity was evaluated on four cancer cell lines (HepG2, HT-29, Hela and BGC-823) by the standard MTT assay, and their nephrotoxicity was tested using MDCK cells. The ClogP values were calculated by means of computer simulation, and logP values of both **6a** and TOA were determined using a shake flask-ultraviolet spectrophotometry method. Among the active compounds, **6a** exhibited promising cytotoxicity, lower toxicity and better hydrophilicity. Combining Giemsa and DAPI staining, it was further verified that **6a** could induce HepG2 apoptosis via nuclei fragmentation and had lower nephrotoxicity than CDDP. The results suggested that the attempt to introduce small, polar or basic amino acids into the hydroxy group of TOA could effectively enhance the hydrophilicity and biological activity. However, it is worthy of further study whether the better activity of TOA-amino acid derivatives is connected with a hydrophilicity increase of the amino acid conjugates, the positive charge, selective identification by membrane surface protein receptors or other ways. Our completed work lays the foundation for further research on the cytotoxicity mechanism, transmembrane transport and bioavailability of TOA-amino acid derivatives.
